# DXA parameters, Trabecular Bone Score (TBS) and Bone Mineral Density (BMD), in fracture risk prediction in endocrine-mediated secondary osteoporosis

**DOI:** 10.1007/s12020-021-02806-x

**Published:** 2021-07-10

**Authors:** Enisa Shevroja, Francesco Pio Cafarelli, Giuseppe Guglielmi, Didier Hans

**Affiliations:** 1 Center of Bone Diseases, Bone & Joint Department, Lausanne University Hospital, Lausanne, Switzerland; 2 Department of Clinical and Experimental Medicine, Foggia University School of Medicine, Foggia, Italy

**Keywords:** Bone mineral density, Dual-energy X-ray absorptiometry, Trabecular bone score, Secondary osteoporosis, Diabetes mellitus, Endocrine disorders

## Abstract

Osteoporosis, a disease characterized by low bone mass and alterations of bone microarchitecture, leading to an increased risk for fragility fractures and, eventually, to fracture; is associated with an excess of mortality, a decrease in quality of life, and co-morbidities. Bone mineral density (BMD), measured by dual X-ray absorptiometry (DXA), has been the gold standard for the diagnosis of osteoporosis. Trabecular bone score (TBS), a textural analysis of the lumbar spine DXA images, is an index of bone microarchitecture. TBS has been robustly shown to predict fractures independently of BMD. In this review, while reporting also results on BMD, we mainly focus on the TBS role in the assessment of bone health in endocrine disorders known to be reflected in bone.

## Background

Osteoporosis is a common disease characterized by low bone mass (i.e. quantity) and altered bone microarchitecture (i.e. quality), resulting in decreased bone strength with an increased risk of fractures [1]. It is associated with an excess of mortality, a decrease in quality of life, and co-morbidities; and has a high social and economic burden, representing a major public health problem. Osteoporosis affects mostly postmenopausal women and fewer men. It is mainly related to the normal aging process— primary osteoporosis. Nevertheless, it can also occur due to secondary causes such as bone-affecting treatments, clinical disorders, or lifestyle habits—secondary osteoporosis [2]. Nine million osteoporotic fractures occur worldwide yearly [3]. The accurate identification of fracture risk, followed by relevant management, would reduce these numbers and the associated costs [4].

Bone mineral density (BMD), measured by dual X-ray absorptiometry (DXA), has been the gold standard for osteoporosis diagnosis in the absence of established fragility fractures [5]. BMD, a bone quantity parameter, is a major determinant of bone strength and fracture risk. Nevertheless, a considerable overlap (up to 45%) exists in BMD values between individuals who develop fractures and those who do not, suggesting that fracture risk prediction based solely on BMD is suboptimal [6]. Thus, the development of bone quality assessments has gained special interest to address this gap. Trabecular bone score (TBS) is one of the most widely used assessments of bone quality [7]. Both BMD and TBS are independent predictors of fragility fractures and are the two pillars of the World Health Organization’s (WHO) clinical definition of osteoporosis [1]. In this article, we review the role of DXA-derived parameters, BMD and TBS, with a special focus on TBS, in fracture risk prediction in endocrine-mediated secondary osteoporosis.

## DXA: the gold standard for BMD assessment

DXA is the most widely used technique and the gold standard for osteoporosis diagnosis and management [8–10]. It is a simple, quick, painless, and safe examination, which uses very low doses of dual X-rays (considerably lower than those of an X-ray and a CT scan), hence it can be repeated safely over time. DXA acquisition can provide images for total body, hip, posterior–anterior (PA) lumbar spine (LS), and/or forearm. BMD (expressed in g/m^2^) is measured in well-defined regions of interest in these images. LS, total hip, femoral neck, and/or 1/3 radius BMD values are considered for the osteoporosis diagnosis and/or management. The decision on the use of each of them depends on factors, such as sex and age of the patient. For example, the PA LS is the preferred region for the BMD assessment in women up to 60 years and in men up to 65; the femoral neck and total hip are the chosen sites for BMD assessment in older individuals and/or in the presence of discrepancies or degenerations in the LS. Forearm (1/3 radius) is a backup site taken into consideration when the main sites (PA LS, total hip, and femoral neck) are impacted by certain health conditions such as hyperparathyroidism or obesity (exceeding the weight limit of the DXA device’s table).

The quantitative assessment of BMD is c*’*ompared with the average BMD value of a population of healthy young adults in the age of peak bone mass (young adult reference population). The number of standard deviations that the BMD differs from the BMD of the young adult reference population is indicated by the term BMD T-score. The WHO classification of osteoporosis is based on the lowest BMD T-score value from PA LS, total hip or femoral neck(or 1/3 radius for the specified scenarios): normal: BMD T-score greater or equal than −1; osteopenia: BMD T-score between −1 and −2.5; osteoporosis: BMD T-score equal or less than −2.5. This classification applies only to the BMD assessed by DXA. Extensive evidence supports the use of DXA BMD for osteoporosis diagnosis, prognosis, and follow-up, for fracture risk stratification and treatment initiation decision-making [11]. The importance of BMD testing, the skeletal sites to measure it, its report and interpretation, and the follow-up intervals between measurements, have been well-defined in several guidelines and taught worldwide by a combined course put together (Osteoporosis Essentials^®^) by the International Osteoporosis Foundation (IOF) and the International Society of Clinical Densitometry (ISCD) [12, 13].

BMD is a measurement of high reliability and precision, able to predict osteoporotic fractures well in advance, and useful in osteoporotic patients‘ follow-up. Black et al. demonstrated that BMD and history of nonvertebral fracture could predict fractures in a period as long as 20–25 years in a large cohort of postmenopausal women [14]. The most widely used tool for fracture prediction, FRAX, provides the fracture probability for a period of 10 years [15]. An extended DXA measurement of the hip diaphysis is recommended in patients on long-term treatment with bisphosphonates in order to detect early signs of atypical femur fractures [16]. ISCD recommends the use of BMD as assessed by DXA for antiosteoporotic treatment follow-up [17].

An acknowledged drawback of DXA is its limited capability to assess bone health in type 2 diabetes mellitus (T2DM) patients [18–20]. Although bone fragility is a known complication of diabetes, T2DM patients have normal or higher BMD levels as compared to non-diabetics. Napoli et al. [21] thoroughly describe the possible mechanisms that could explain this association: in vivo and ex vivo studies have shown the anabolic effect of insulin on osteoblasts, thus hyperinsulinemia in patients with T2DM might explain the high BMD levels; also, sclerostin levels are higher among T2DM and they are positively associated with BMD. Despite the normal or higher BMD values in diabetics as compared to non-diabetics, several studies [22–24] have shown that low BMD values are associated with a higher risk of fracture in diabetics as compared to non-diabetics. Moreover, BMD is useful at antiosteoporotic treatment follow-up in diabetic patients [22–25]. Other limitations of DXA are the overestimation of LS BMD as a consequence of degenerative changes in the spine or aortic calcification presence, and the influence of BMD by BMI or regional soft tissue presence [26, 27]. Armameto-Villareal et al. [28] has recently shown that weight loss among obese individuals is associated with bone loss as indicated by BMD values. However, BMD was negatively associated with the presence of fat tissue, indicating that the increase in body fat may increase systemic inflammation leading to frailty and poor bone quality. To address the increase in bone loss and eventual fracture risk in obese undergoing weight loss therapy, Armameto-Villareal et al. suggest a combination of aerobic and resistance exercise and calcium and vitamin D supplements. Other bone health assessments using bone density derived from high-resolution peripheral quantitative computed tomography (HR-pQCT) or other bone strength surrogates (such as bone turnover markers or advanced glycation end-products, etc.) in obese and diabetics have been shown useful at their overall bone health evaluation [29–31].

Major improvements in DXA technologies over the years have enabled the assessment of bone health parameters other than BMD. For example, the improved spatial resolution (as low as 250 micrometers for some of DXA devices) allows the search for vertebral fractures (vertebral fracture assessment—VFA) and the assessment of trabecular bone texture (TBS) (further elaborated below). Different algorithms can also estimate structural parameters of hip geometry, such as femoral strength index, axis length, cross-sectional area, etc. (hip structural analysis—HSA) [32–35].

## DXA: beyond bone health assessment

Besides bone, DXA assesses parameters of muscle and fat [36]. Several authors reported the direct and indirect role of DXA in the follow-up of malabsorption conditions [37–40]. In addition, recently, emerged more and more the key concept of the “modified functional muscle–bone unit”, in which BMD is strongly associated with either mass and quality of muscles, which are further correlated with fracture risk [41]. In this sense, DXA has been demonstrated useful in providing a precise assessment of the skeletal muscle mass, which is crucial in disorders such as sarcopenia or geriatric syndrome [42, 43]. Other parameters of body composition, indicating the presence and distribution of fat mass in different regions of the body are essential in the assessment and follow-up of health disorders associated with fat presence [44–48].

An interesting recent study demonstrated that during weight-loss programs, BMI can not specify the fat distribution, with limited use of this datum; in contrast with DXA report, which is able to quantify the metabolic re-distribution of total and regional fat mass and visceral adipose tissue [49]. Another study compared the accuracy of six different osteoporosis risk assessment tools in a restricted group of women in Kuala Lumpur, further demosntrating the importance of DXA in such context [50].

## Definition of TBS and its clinical validation/implication

**Fig. 1:**
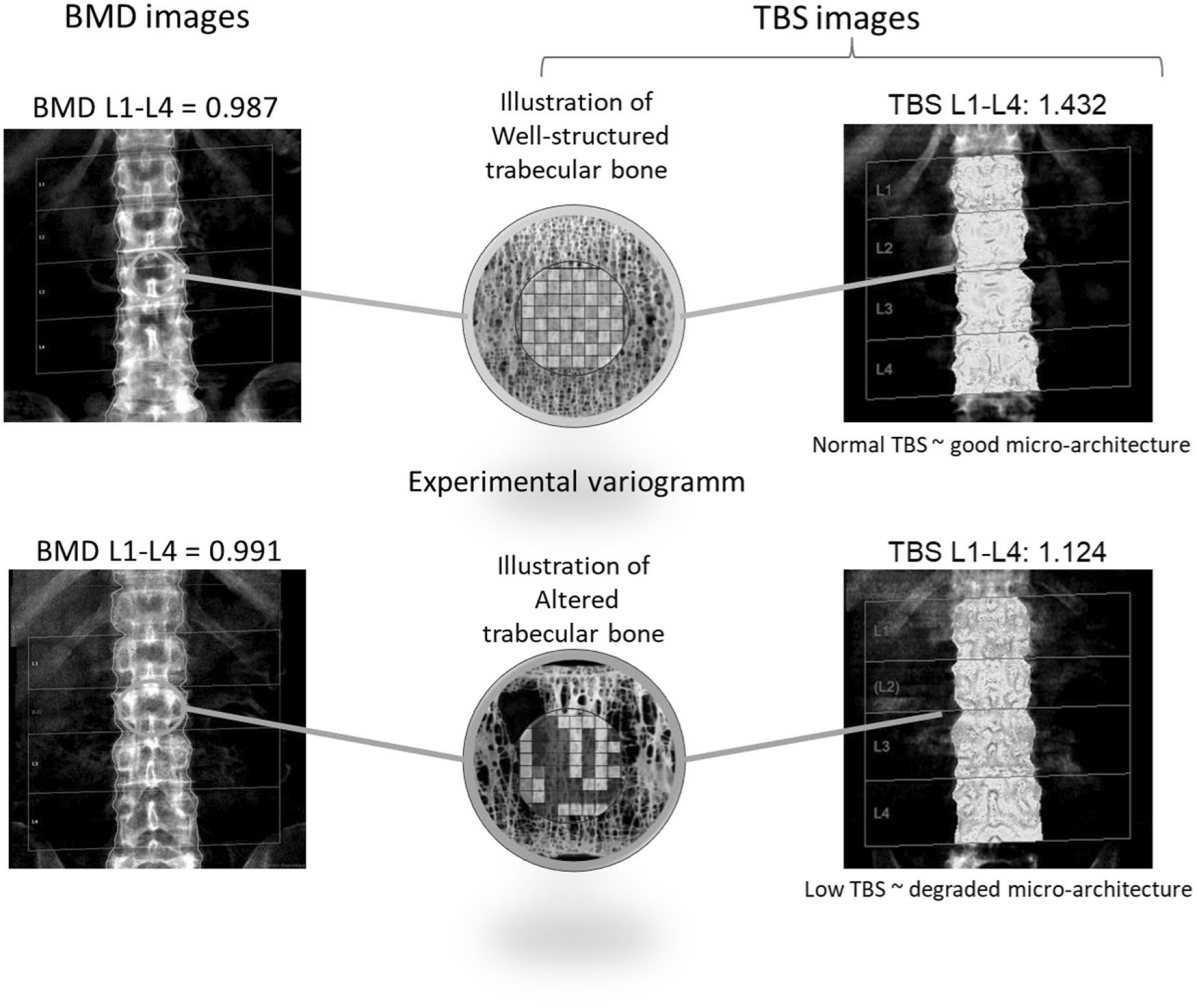
Concept of TBS. Two different patients with equivalent bone mineral density (BMD) but different trabecular bone score (TBS)

TBS is a textural analysis resulting from a computed evaluation of pixel gray-level variations in previously obtained LS DXA images. It is an index of bone microarchitecture correlated with parameters of bone strength [51–53]. Higher values of TBS indicate a better microarchitecture, whereas lower values indicate a degraded microarchitecture (Fig. 1). Studies have robustly shown that TBS predicts fractures independently of BMD and other clinical risk factors for fracture. The added value of the TBS to BMD in fracture risk assessment has been extensively documented in cross-sectional, prospective, and longitudinal studies [54] and endorsed by medical societies of bone field (IOF, the European Society for Clinical and Economic Aspects of Osteoporosis and Osteoarthritis, and the ISCD) [55]. More recently, for regions where intervention guidelines are based solely on BMD T-score, an alternative approach for using TBS in clinical practice based upon a “risk-equivalent” offset adjustment to BMD T-score has been developed [56]. The early validations of this approach illustrate how TBS contributes to vertebral fracture risk assessment by increasing the specificity of the model (by about 20%) without compromising the sensitivity.

**Fig. 2:**
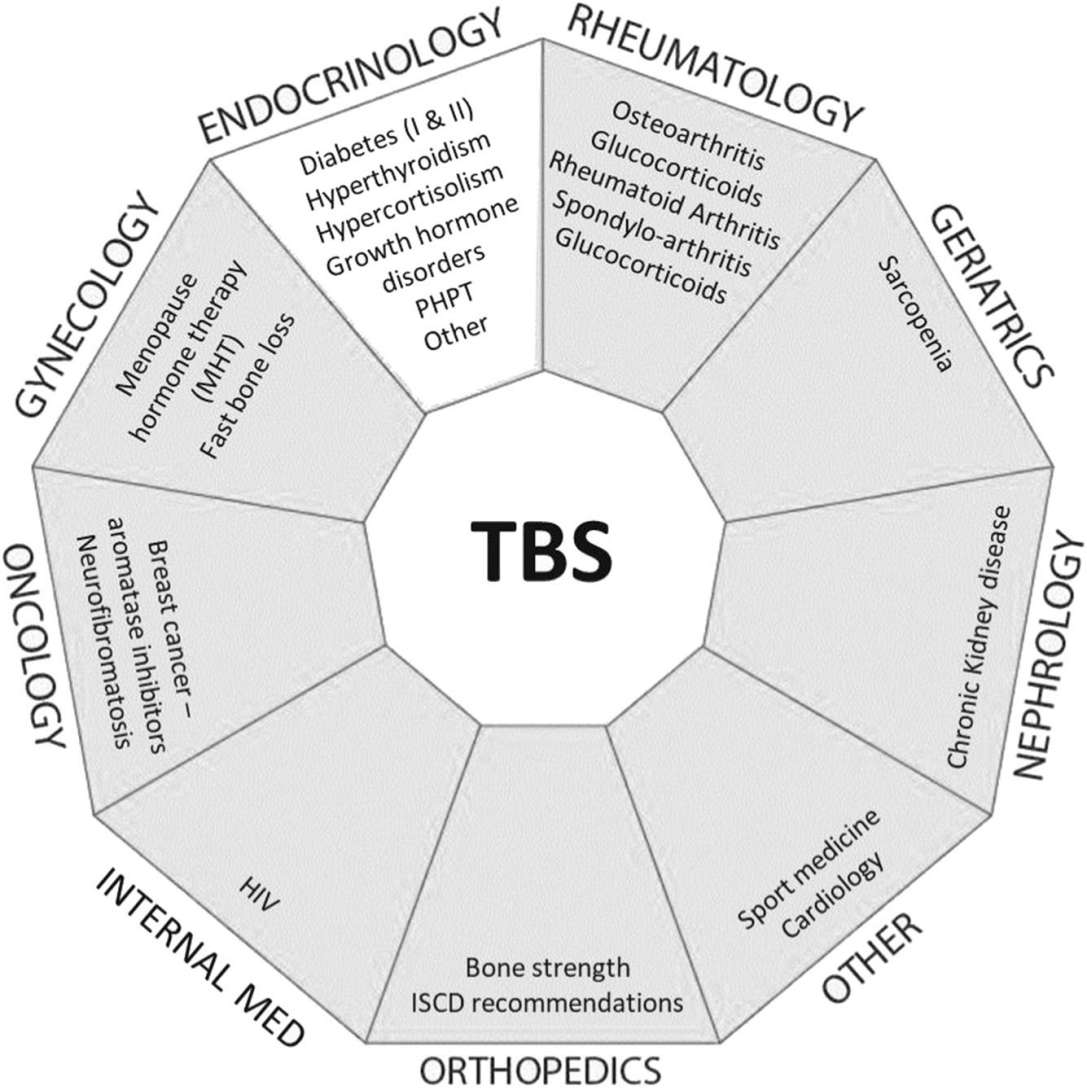
Summary of pathologies (per medical specialty) in which TBS has demonstrated added clinical value (published studies only)—adapted to this paper needs—Courtesy of Medimaps Group SA (Switzerland)

Several studies have also demonstrated the ability of TBS to predict fragility fractures in secondary osteoporosis caused by diabetes, PHPT, rheumatoid arthritis, adrenal incidentaloma, chronic kidney disease, long-term glucocorticoid therapy, HIV, or oncological conditions (Fig. 2) [2].

## TBS in endocrine-mediated secondary osteoporosis

### TBS and diabetes mellitus

It is well established that the skeleton is one of the organs affected by diabetes mellitus type 1 and 2, listing diabetes as one of the risk factors for fragility fractures [57]. Despite the high fracture risk in diabetics, their BMD is generally higher compared to non-diabetics. Thus, it is hypothesized that diabetes may be associated with a reduction of bone strength that is not reflected in the measurement of BMD alone. Robust evidence has shown that—differently than BMD—TBS is lower in diabetics than non-diabetics [16, 17, 19, 57]. In 2013, Leslie et al. were the first to report this finding in a study of 29,000 women, of which 2,356 had diabetes [58]. Moreover, it has been confirmed that TBS is negatively related to levels of HbA1c (e.g. bone structure remained normal when HbA1c was lower than 7.5), fasting plasma glucose, and fasting insulin [59, 60]. Currently, several studies conducted in more than 40,508 (4,269 diabetics) individuals altogether have robustly shown that TBS is lower in diabetics than in controls, whereas BMD is higher in diabetics than in controls [58–70]. Furthermore, TBS outperforms BMD in fracture discrimination and prediction in diabetics. A recent meta-analysis of 7,819 women and men also showed that type 2 diabetes was associated with decreased TBS in fully adjusted models; and that compared to controls, also prediabetics had significantly lower TBS [71]. Overall, combining TBS and BMD incrementally improves fracture prediction. Nevertheless, the increased fracture risk is not entirely explained by the TBS difference between the diabetics and non-diabetics: diabetes is associated with a 32% increase in the fracture risk; whereas one standard deviation decrease in TBS is associated with 1.27-fold (95% CI 1.10–1.46) increase in the risk of fracture in diabetics [72]. The mechanism remains unclear. A hypothesis to explain the association of TBS with diabetes is that advanced glycation end products could mediate this association [61]. Nevertheless, it necessitates further investigation to be proven true.

### TBS and growth hormone (GH) disorders

GH is an important regulator of bone growth; its effects are important to maintain bone mass [73]. *Growth hormone deficiency* (GHD) is related to reduced bone strength and the GH long-term replacement therapy can successfully revert this condition [68]. The effect of GH treatment on bone quality is unclear. Kužma et al. showed that bone quality, as assessed by TBS, improved only in the GHD cases with sufficient vitamin D levels. However, an insignificant decrease in TBS was seen after 7 years of GH treatment [74]. *Acromegaly* is a rare disease characterized by excessive GH and insulin-like growth factor 1 (IGF-1), caused mainly by GH-producing pituitary adenoma [75]. Evidence shows that patients with acromegaly are predisposed to develop fractures regardless of their BMD values. To explain the high incidence of fractures in these patients, TBS may be useful in assessing skeletal fragility, given BMD is unable to accurately assess bone strength. Previous studies suggested that GH excess may positively affect cortical bone and negatively affect trabecular bone [74]. Currently, there are conflicting BMD results in acromegaly, showing mainly no effect or increase. Further, acromegaly treatment had different effects on TBS and BMD: TBS decreased significantly, whereas BMD increased [74].

### TBS in hyperparathyroidism

*Primary hyperparathyroidism* (PHPT) is an endocrine disorder characterized by elevated parathyroid hormone levels with hypercalcemia. Patients with PHPT exhibit increased fracture risk [76]. Several studies have reported that PHPT cases have decreased TBS, indicating deteriorated bone microarchitecture. TBS is shown to be a predictor of fracture independent of BMD in PHPT patients. Also, the fractured patients with PHPT had lower TBS than the non-fractured ones [77–80]. Moreover, the TBS values significantly improved after parathyroidectomy compared to after conservative management of PHPT [81].

### TBS in hyperthyroidism

Thyroid hormone stimulates bone resorption. Hyperthyroidism is associated with bone loss and increased risk for fracture. A negative association between bone parameters and the fT4 level has been reported by Hwangbo et al. [82]. Moreover, TBS was studied in thyroid carcinoma patients receiving long-term *thyroid-stimulating hormone suppressive therapy*, and resulted to be lower than in patients in shorter-term therapy [83, 84]. *Grave’s disease*, an autoimmune disorder that causes hyperthyroidism, has been shown to have a strong correlation with decreased TBS, whereas BMD did not differ between the cases and control groups [85]. The findings of the studies on TBS and health conditions exhibited with hyperthyroidism are in line with each other and bring evidence that TBS is a bone parameter that aids in the management of patients suffering from this thyroid disorder.

### TBS in hypercortisolism

*Cushing’s disease* (CD) is characterized by an increased level of cortisol, namely hypercortisolism; and is an endocrine cause of obesity [86]. It has been shown that hypercortisolism has a negative effect on bone due to decreased bone formation that it causes. Nevertheless, only a mild decline in BMD has been reported; whereas the TBS decline was more pronounced [87, 88]. Moreover, TBS was lower in CD than in primary obesity patients, indicating the sensitivity of TBS to detect the variations along the hypercortisolism spectrum. Eller-Vainicher et al. investigated TBS in *subclinical hypercortisolism*; and demonstrated that the TBS decrease was associated with the cortisol excess and the severity of fractures and that TBS was more accurate than BMD in identifying patients at high risk for fracture [89]. Interestingly, Gonzalez Rodriguez et al. concluded in their study that high values of *evening cortisol* were associated with low TBS and increased prevalence of fracture in healthy postmenopausal women [90]. This evidence suggests the utility of TBS in bone health assessment in the spectrum of cortisol disorders.

### TBS and other endocrine disorders

Few efforts have been devoted to investigating TBS in *hypogonadism* conditions, such as *Klinefelter Syndrome* or *estrogen deprivation*. TBS did not change significantly between men with the Klinefelter Syndrome than in controls; whereas BMD was lower in cases [91]. In estrogen deprivation conditions, the decrease of TBS was more than the decrease of BMD, suggesting its independent role in bone health assessment and fracture prediction in these individuals [92].

*Primary aldosteronism* (PA), characterized by the hypersecretion of aldosterone, is associated with high fracture risk; a fact lacking support from studies assessing the association between PA and BMD. Kim et al. studied the values of TBS and BMD in cases of PA versus controls and showed that TBS was significantly lower among cases than controls, whereas BMD did not change significantly between the two groups [93]. PA might represent another endocrine disorder where TBS outperforms BMD in the assessment of skeletal fragility and the explanation of the high risk of fracture.

### TBS in obesity

Inconsistent evidence exists on the presence of fracture risk and bone health condition in obesity. In general, BMD has shown to be higher among obese, while TBS lower. The difference in their associations with BMI has mainly been addressed to the possible technical limitation of TBS. However, the impact of overlying soft tissue on the measurement site is not only a matter of TBS. It is also problematic for BMD [94]. Nevertheless, to account for such limitation in obese, TBS is not applied in individuals with a BMI > 37 kg/m^2^. Furthermore, for a deeper understanding and solution-seeking of this issue, a correction for direct regional soft tissue thickness, taking into account the morphotype of the patients had recently been developed to the TBS algorithm and has shown to surpass this limitation [95]. Further investigations of the updated—in regards to the soft tissue adjustment—TBS algorithm are necessary to support this initial evidence [96].

## Conclusion

Alterations of bone strength in certain endocrine disorders are not always reflected in the BMD values, as their cause may lie in the microarchitecture of the bone. The TBS utility in fracture risk assessment in primary osteoporosis and diabetes has been robustly established. However, further larger studies could help for a deeper insight into the TBS behavior in other endocrine disorders where the presence of increased fracture risk is not (or only partially) explained by the BMD values, while TBS alone or in combination with BMD have proved promising at explaining the increased fracture risk.

In summary, knowledge of bone microarchitecture enrichens our understanding of the pathophysiology of primary and secondary osteoporosis. Nevertheless, bone quality cannot be comprehensively characterized by one sole parameter. Current noninvasive imaging techniques in combination with ex vivo mechanical and compositional techniques might provide a thorough understanding of bone quality. Currently, integrating the combined use of BMD, TBS, and clinical risk factors in clinical routine have proven efficient for osteoporosis and fracture risk management. This combination—within FRAX or not—enables us to fine-tune the risk of fracture stratification, treatment decision, and disease management.

## References

[1] Consensus Development Conference (1993). Diagnosis, prophylaxis, and treatment of osteoporosis. Am. J. Med..

[2] Ulivieri FM, Silva BC, Sardanelli F, Hans D, Bilezikian JP, Caudarella R (2014). Utility of the trabecular bone score (TBS) in secondary osteoporosis. Endocrine.

[3] Johnell O, Kanis JA (2006). An estimate of the worldwide prevalence and disability associated with osteoporotic fractures. Osteoporos. Int..

[4] R.B. Conley et al. J. Bone Miner. Res. **35**(1), 36–52 (2020). 10.1002/jbmr.3877 31538675

[5] Blake GM, Fogelman I (2009). The clinical role of dual energy X-ray absorptiometry. Eur. J. Radiol..

[6] Marshall D, Johnell O, Wedel H (1996). Meta-analysis of how well measures of bone mineral density predict occurrence of osteoporotic fractures. BMJ.

[7] Hans D, Goertzen AL, Krieg M-A, Leslie WD (2011). Bone microarchitecture assessed by TBS predicts osteoporotic fractures independent of bone density: the Manitoba study. J. Bone Miner. Res..

[8] Messina C, Bignotti B, Bazzocchi A (2017). A critical appraisal of the quality of adult dual-energy X-ray absorptiometry guidelines in osteoporosis using the AGREE II tool: an EuroAIM initiative. Insights Imaging.

[9] Woo T (2019). Radiographic/MR imaging correlation of spinal bony outlines magnetic resonance imaging clinics. Magn Reson Imaging Clin N Am..

[10] Kanis JA, McCloskey EV, Johansson H (2013). European guidance for the diagnosis and management of osteoporosis in postmenopausal women. Osteoporos. Int..

[11] Guglielmi G, Nasuto M (2016). Metabolic bone disease: an updated view—part one. Semin. Musculoskelet. Radiol..

[12] Messina C, Sconfienza L, Bandirali M, Guglielmi G, Ulivieri F (2016). Adult dual-energy X-ray absorptiometry in clinical practice: how I report it. Semin. Musculoskelet. Radiol..

[13] Conley RB, Adib G, Adler RA (2020). Secondary fracture prevention: consensus clinical recommendations from a multistakeholder coalition. J. Bone Miner. Res..

[14] Black DM, Cauley JA, Wagman R (2018). The ability of a single BMD and fracture history assessment to predict fracture over 25 years in postmenopausal women: the study of osteoporotic fractures. J. Bone Miner. Res..

[15] Kanis JA, Harvey NC, Johansson H, Odén A, Leslie WD, McCloskey EV (2017). FRAX update. J. Clin. Densitom..

[16] Black DM, Abrahamsen B, Bouxsein ML, Einhorn T, Napoli N (2019). Atypical femur fractures: review of epidemiology, relationship to bisphosphonates, prevention, and clinical management. Endocr. Rev..

[17] Shuhart CR, Yeap SS, Anderson PA (2019). Executive summary of the 2019 ISCD position development conference on monitoring treatment, DXA cross-calibration and least significant change, spinal cord injury, peri-prosthetic and orthopedic bone health, transgender medicine, and pediatrics. J. Clin. Densitom..

[18] Poiana C, Capatina C (2019). Osteoporosis and fracture risk in patients with type 2 diabetes mellitus. Acta Endocrinol..

[19] Napoli N, Conte C, Pedone C (2019). Effect of insulin resistance on BMD and fracture risk in older adults. J. Clin. Endocrinol. Metab..

[20] Napoli N, Schwartz AV, Schafer AL (2018). Osteoporotic fractures in men (MrOS) Study Research Group. Vertebral fracture risk in diabetic elderly men: the MrOS study. J. Bone Miner. Res..

[21] Napoli N, Strollo R, Paladini A, Briganti SI, Pozzilli P, Epstein S (2014). The alliance of mesenchymal stem cells, bone, and diabetes. Int. J. Endocrinol..

[22] Schwartz AV, Vittinghoff E, Bauer DC (2011). Study of Osteoporotic Fractures (SOF) Research Group; Osteoporotic Fractures in Men (MrOS) Research Group; Health, Aging, and Body Composition (Health ABC) Research Group. Association of BMD and FRAX score with risk of fracture in older adults with type 2 diabetes. JAMA.

[23] Villareal DT, Chode S, Parimi N (2011). Weight loss, exercise, or both and physical function in obese older adults. N. Engl. J. Med..

[24] Armamento-Villareal R, Sadler C, Napoli N (2012). Weight loss in obese older adults increases serum sclerostin and impairs hip geometry but both are prevented by exercise training. J. Bone Miner. Res..

[25] Napoli N, Strotmeyer ES, Ensrud KE (2014). Fracture risk in diabetic elderly men: the MrOS study. Diabetologia.

[26] Kinoshita H, Tamaki T, Hashimoto T, Kasagi F (1998). Factors influencing lumbar spine bone mineral density assessment by dual-energy X-ray absorptiometry: comparison with lumbar spinal radiogram. J. Orthop. Sci..

[27] Setiawati R, Di Chio F, Rahardjo P, Nasuto M, Dimpudus FJ, Guglielmi G (2016). Quantitative assessment of abdominal aortic calcifications using lateral lumbar radiograph, dual-energy X-ray absorptiometry, and quantitative computed tomography of the spine. J. Clin. Densitom..

[28] Armamento-Villareal R, Aguirre L, Waters DL, Napoli N, Qualls C, Villareal DT (2020). Effect of aerobic or resistance exercise, or both, on bone mineral density and bone metabolism in obese older adults while dieting: a randomized controlled trial. J. Bone Miner. Res..

[29] Vigevano F, Gregori G, Colleluori G (2021). In men with obesity, T2DM Is associated with poor trabecular microarchitecture and bone strength and low bone turnover. J. Clin. Endocrinol. Metab..

[30] G. Colleluori, L. Aguirre, N. Napoli, C. Qualls, D.T. Villareal, R. Armamento-Villareal. Testosterone therapy effects on bone mass and turnover in hypogonadal men with type 2 diabetes. J. Clin. Endocrinol. Metab. dgab18. 10.1210/clinem/dgab181 (2021). Epub ahead of print 10.1210/clinem/dgab181 PMC8599870 33735389

[31] Piccoli A, Cannata F, Strollo R (2020). Sclerostin regulation, microarchitecture, and advanced glycation end-products in the bone of elderly women with type 2 diabetes. J. Bone Miner. Res..

[32] Guglielmi S, Muscarella A (2011). Bazzocchi, Integrated imaging approach to osteoporosis: state-of-the-art review and update. Radiographics.

[33] Andreoli G, Scalzo G, Masala S, Tarantino U, Guglielmi G (2009). Body composition assessment by dual-energy X-ray absorptiometry (DXA). Radiol. Med..

[34] Bonaccorsi G, Cafarelli FP, Cervellati C (2020). A new corrective model to evaluate TBS in obese post-menopausal women: a cross-sectional study. Aging Clin. Exp. Res..

[35] Messina C, Lastella G, Sorce S, Piodi LP, Rodari G, Giavoli C, Marchelli D, Guglielmi G, Ulivieri FM (2018). Pediatric dual-energy X-ray absorptiometry in clinical practice: what the clinicians need to know. Eur. J. Radiol..

[36] G. Guglielmi, Editorial. Eur. J. Radiol. 85, 1453–1455 (2016) 10.1016/j.ejrad.2016.05.011 27368763

[37] Andreoli A, Garaci F, Cafarelli FP, Guglielmi G (2016). Body composition in clinical practice. Eur. J. Radiol..

[38] N. Napoli, Am. J. Clin. Nutr. 85 (5), 1428–1433 (2007) 10.1093/ajcn/85.5.1428 PMC9087302 17490982

[39] Bellanti F, Romano AD, Lo Buglio A, Castriotta V, Guglielmi G, Greco A, Serviddio G, Vendemiale G (2018). Oxidative stress is increased in sarcopenia and associated with cardiovascular disease risk in sarcopenic obesity. Maturitas.

[40] Phan CM, Guglielmi G (2016). Metabolic bone disease in patients with malabsorption. Semin. Musculoskelet. Radiol..

[41] Zhang Y, Guo J, Duanmu Y (2019). Quantitative analysis of modified functional muscle-bone unit and back muscle density in patients with lumbar vertebral fracture in Chinese elderly men: a case-control study. Aging Clin. Exp. Res..

[42] Messina C, Maffi G, Vitale JA, Ulivieri FM, Guglielmi G, Sconfienza LM (2018). Diagnostic imaging of osteoporosis and sarcopenia: a narrative review. Quant. Imaging Med. Surg..

[43] Carnevale V, Castriotta V, Piscitelli PA (2018). Assessment of skeletal muscle mass in older people: comparison between 2 anthropometry-based methods and dual-energy X-ray absorptiometry. J. Am. Med. Dir. Assoc..

[44] Armamento-Villareal R, Aguirre L, Napoli N (2014). Changes in thigh muscle volume predict bone mineral density response to lifestyle therapy in frail, obese older adults. Osteoporos. Int..

[45] Guglielmi G, Ponti F, Agostini M, Amadori M, Battista G, Bazzocchi A (2016). The role of DXA in sarcopenia. Aging Clin. Exp. Res..

[46] Ponti F, Plazzi A, Guglielmi G, Marchesini G, Bazzocchi A (2019). Body composition, dual-energy X-ray absorptiometry and obesity: the paradigm of fat (re)distribution. BJR Case Rep..

[47] A. Iolascon, J. Endocrinol. Investig. 34 (1), e12–e15 (2011) 10.1007/BF03346703 20634640

[48] Bonaccorsi G, Trentini A, Greco P (2019). Changes in adipose tissue distribution and association between uric acid and bone health during menopause transition. Int. J. Mol. Sci..

[49] Ponti F, Soverini V, Plazzi A (2018). DXA-assessed changes in body composition in obese women following two different weight loss programs. Nutrition.

[50] Toh LS, Lai PSM, Wu DB, Bell BG, Dang CPL, Low BY, Wong KT, Guglielmi G, Anderson C (2019). A comparison of 6 osteoporosis risk assessment tools among postmenopausal women in Kuala Lumpur, Malaysia. Osteoporos. Sarcopenia.

[51] Baldini M, Grossi E, Cappellini MD (2017). The role of trabecular bone score and hip geometry in Thalassemia Major: a neural network analysis. Br. J. Res..

[52] Bazzocchi A, Ponti F, Diano D, Amadori M, Albisinni U, Battista G (2015). Trabecular bone score in healthy ageing. Br. J. Radiol..

[53] Pothuaud L, Carceller P, Hans D (2008). Correlations between grey-level variations in 2D projection images (TBS) and 3D microarchitecture: applications in the study of human trabecular bone microarchitecture. Bone.

[54] Shevroja E, Lamy O, Kohlmeier L, Koromani F, Rivadeneira F, Hans D (2017). Use of Trabecular Bone Score (TBS) as a complementary approach to Dual-energy X-ray Absorptiometry (DXA) for fracture risk assessment in clinical practice. J. Clin. Densitom..

[55] Harvey NC, Glüer CC, Binkley N (2015). Trabecular bone score (TBS) as a new complementary approach for osteoporosis evaluation in clinical practice. Bone.

[56] Leslie WD, Shevroja E, Johansson H (2018). Risk-equivalent T-score adjustment for using lumbar spine trabecular bone score (TBS): the Manitoba BMD registry. Osteoporos. Int..

[57] Jiang N, Xia W (2018). Assessment of bone quality in patients with diabetes mellitus. Osteoporos. Int..

[58] Leslie WD, Aubry-Rozier B, Lamy O, Hans D (2013). Manitoba bone density P. TBS (trabecular bone score) and diabetes-related fracture risk. J. Clin. Endocrinol. Metab..

[59] Dhaliwal R, Cibula D, Ghosh C, Weinstock RS, Moses AM (2014). Bone quality assessment in type 2 diabetes mellitus. Osteoporos. Int..

[60] Kim JH, Choi HJ, Ku EJ (2015). Trabecular bone score as an indicator for skeletal deterioration in diabetes. J. Clin. Endocrinol. Metab..

[61] Ho-Pham LT, Nguyen TV (2019). Association between trabecular bone score and type 2 diabetes: a quantitative update of evidence. Osteoporos. Int..

[62] Zhukouskaya VV, Eller-Vainicher C, Gaudio A (2016). The utility of lumbar spine trabecular bone score and femoral neck bone mineral density for identifying asymptomatic vertebral fractures in well-compensated type 2 diabetic patients. Osteoporos. Int..

[63] Bonaccorsi G, Fila E, Messina C (2017). Comparison of trabecular bone score and hip structural analysis with FRAX(®) in postmenopausal women with type 2 diabetes mellitus. Aging Clin. Exp. Res..

[64] Xue Y, Baker AL, Nader S (2018). Lumbar spine trabecular bone score (TBS) reflects diminished bone quality in patients with diabetes mellitus and oral glucocorticoid therapy. J. Clin. Densitom..

[65] Caffarelli C, Giambelluca A, Ghini V (2017). In Type-2 diabetes subjects trabecular bone score is better associated with carotid intima-media thickness than BMD. Calcif. Tissue Int..

[66] A. Alois Karl Wagner. Trabecular bone score in non-diabetic, prediabetic and type II diabetic subjects. MD thesis, Medical University of Graz, Graz, Austria, (2017)

[67] Iki M, Fujita Y, Kouda K (2017). Hyperglycemia is associated with increased bone mineral density and decreased trabecular bone score in elderly Japanese men: The Fujiwara-kyo osteoporosis risk in men (FORMEN) study. Bone.

[68] Holloway KL, De Abreu LLF, Hans D (2018). Trabecular bone score in men and women with impaired fasting glucose and diabetes. Calcif. Tissue Int..

[69] Rianon N, Ambrose CG, Buni M (2018). Trabecular bone score is a valuable addition to bone mineral density for bone quality assessment in older Mexican American Women with Type 2 diabetes. J. Clin. Densitom..

[70] Ho-Pham LT, Tran B, Do AT, Nguyen TV (2019). Association between pre-diabetes, type 2 diabetes and trabecular bone score: the Vietnam Osteoporosis Study. Diabetes Res. Clin. Pract..

[71] Koromani F, Oei L, Shevroja E (2020). Vertebral fractures in individuals with Type 2 diabetes: more than skeletal complications alone. Diabetes Care.

[72] Rubin MR, Patsch JM (2016). Assessment of bone turnover and bone quality in type 2 diabetic bone disease: current concepts and future directions. Bone Res..

[73] Kužma M, Killinger Z, Jackuliak P (2019). Pathophysiology of growth hormone secretion disorders and their impact on bone microstructure as measured by trabecular bone score. Physiol. Res..

[74] Kužma M, Kužmová Z, Zelinková Z (2014). Impact of the growth hormone replacement on bone status in growth hormone deficient adults. Growth Horm. IGF Res..

[75] Kasuki L, Rocha PDS, Lamback EB, Gadelha MR (2019). Determinants of morbidities and mortality in acromegaly. Arch. Endocrinol. Metab..

[76] Silva BC, Broy SB, Boutroy S, Schousboe JT, Shepherd JA, Leslie WD (2015). Fracture risk prediction by non-BMD DXA measures: the 2015 ISCD Official Positions Part 2: Trabecular Bone Score. J. Clin. Densitom..

[77] Romagnoli E, Cipriani C, Nofroni I (2013). “Trabecular Bone Score” (TBS): an indirect measure of bone micro-architecture in postmenopausal patients with primary hyperparathyroidism. Bone.

[78] Eller-Vainicher C, Filopanti M, Palmieri S (2013). Bone quality, as measured by trabecular bone score, in patients with primary hyperparathyroidism. Eur. J. Endocrinol..

[79] Hong AR, Lee JH, Kim JH, Kim SW, Shin CS (2019). Effect of endogenous parathyroid hormone on bone geometry and skeletal microarchitecture. Calcif. Tissue Int..

[80] Muñoz-Torres M, Manzanares Córdova R, García-Martín A (2019). Usefulness of Trabecular Bone Score (TBS) to identify bone fragility in patients with primary hyperparathyroidism. J. Clin. Densitom..

[81] Cipriani C, Abraham A, Silva BC (2017). Skeletal changes after restoration of the euparathyroid state in patients with hypoparathyroidism and primary hyperparathyroidism. Endocrine.

[82] Hwangbo Y, Kim JH, Kim SW (2016). High-normal free thyroxine levels are associated with low trabecular bone scores in euthyroid postmenopausal women. Osteoporos. Int..

[83] De Mingo Dominguez ML, Guadalix Iglesias S, Martin-Arriscado Arroba C, López Alvarez B, Martínez Diaz-Guerra G, Martinez-Pueyo JI (2018). Low trabecular bone score in postmenopausal women with differentiated thyroid carcinoma after long-term TSH suppressive therapy. Endocrine.

[84] Moon JH, Kim KM, Oh TJ, Choi SH, Lim S, Park YJ (2017). The effect of TSH suppression on vertebral trabecular bone scores in patients with differentiated thyroid carcinoma. J. Clin. Endocrinol. Metab..

[85] Kužma M, Vaňuga P, Binkley N, Ságová I, Pávai D, Blažíček P (2018). High serum fractalkine is associated with lower trabecular bone score in premenopausal women with Graves’ disease. Horm. Metab. Res..

[86] Pivonello R, De Leo M, Cozzolino A, Colao A (2015). The treatment of Cushing’s disease. Endocr. Rev..

[87] Belaya ZE, Hans D, Rozhinskaya LY, Dragunova NV, Sasonova NI, Solodovnikov AG (2015). The risk factors for fractures and trabecular bone-score value in patients with endogenous Cushing’s syndrome. Arch. Osteoporos..

[88] Batista SL, de Araújo IM, Carvalho AL, Alencar MAVSD, Nahas AK, Elias J (2019). Beyond the metabolic syndrome: visceral and marrow adipose tissues impair bone quantity and quality in Cushing’s disease. PLoS ONE.

[89] Eller-Vainicher C, Morelli V, Ulivieri FM, Palmieri S, Zhukouskaya VV, Cairoli E (2012). Bone quality, as measured by trabecular bone score in patients with adrenal incidentalomas with and without subclinical hypercortisolism. J. Bone Miner. Res..

[90] Gonzalez Rodriguez E, Lamy O, Stoll D, Metzger M, Preisig M, Kuehner C (2017). High evening cortisol level is associated with low TBS and increased prevalent vertebral fractures: OsteoLaus study. J. Clin. Endocrinol. Metab..

[91] Tahani N, Nieddu L, Prossomariti G, Spaziani M, Granato S, Carlomagno F (2018). Long-term effect of testosterone replacement therapy on bone in hypogonadal men with Klinefelter Syndrome. Endocrine.

[92] Pedrazzoni M, Casola A, Verzicco I, Abbate B, Vescovini R, Sansoni P (2014). Longitudinal changes of trabecular bone score after estrogen deprivation: effect of menopause and aromatase inhibition. J. Endocrinol. Investig..

[93] Kim B-J, Kwak MK, Ahn SH, Kim H, Lee SH, Koh J-M (2018). Lower trabecular bone score in patients with primary aldosteronism: human skeletal deterioration by aldosterone excess. J. Clin. Endocrinol. Metab..

[94] Amnuaywattakorn S, Sritara C, Utamakul C, Chamroonrat W, Kositwattanarerk A, Thamnirat K (2016). Simulated increased soft tissue thickness artefactually decreases trabecular bone score: a phantom study. BMC Musculoskelet. Disord..

[95] Shevroja E, Aubry-Rozier B, Hans G, Rodriguez EG, Stoll D, Lamy O (2019). Clinical performance of the updated trabecular bone score (TBS) Algorithm, which accounts for the soft tissue thickness: the OsteoLaus Study. J. Bone Miner. Res..

[96] Bazzocchi A, Ponti F, Albisinni U, Battista G, Guglielmi G (2016). DXA: technical aspects and application. Eur. J. Radiol..

